# Proprioception in Above-the-Knee Amputees with Artificial Limbs

**DOI:** 10.1155/2013/417982

**Published:** 2013-11-13

**Authors:** E. P. Latanioti, A. G. Angoules, E. C. Boutsikari

**Affiliations:** ^1^Asclepeion Voulas Hospital, Department of Physical Therapy, Athens, Greece; ^2^General Department of Essential Medical Subjects, Faculty of Health & Caring Professions, Technological Educational Institute, Athens, Greece; ^3^Physical Therapy Department, Faculty of Health & Caring Professions, Technological Educational Institute, Athens, Greece

## Abstract

*Purpose*. To evaluate the lower limb proprioceptive sensation in patients with femoral amputation who received an artificial joint. *Materials and Methods*. 22 patients (18 men, 4 women), 24–65 years old (mean: 42), who had undergone above-the-knee joint amputation and underwent evaluation of proprioception using joint reposition in a predetermined angle of 15° knee flexion. The measurements were applied using a conventional goniometer to both amputated and healthy knees. The last ones were used as internal control. All patients performed an active knee flexion from hyperextension to 15° in a closed kinetic chain in order to evaluate proprioceptive sensation of the knee joint using the joint position sense (JPS) method during specific controllable circumstances very close to normal gait. *Results*. JPS at 15° flexion for the amputated knee was calculated to be equal to 13.91 (SD = ±4.74), and for the healthy side it was equal to 14.15 (SD = ±2.61). No statistically significant differences were detected between the amputated and the healthy limb (*P* > 0.05). *Conclusions*. The proprioceptive information of the stumps did not appear to be affected significantly after thigh amputation and application of artificial prosthesis when JPS at 15° was evaluated. It seems that these patients compensate the loss of the knee sensory receptors via alternative mechanisms.

## 1. Introduction

Amputation of lower limb causes a series of changes and concomitant adjustments which are related not only to the mutilated limb but also to the entire body [1–4]. The sensorimotor system plays major role in these adjustments, as it includes all the centripetal and centrifugal pathways required for the control of posture and movement and the final control of the joint functionality [5–7].

 The sensorimotor system with its complex mechanisms includes the term of proprioception according to an earlier definition. This intricate process involves the transport of information to the central nervous system, relative to the sense of joint position in space, the feeling of power that develops in the joint (sense of force) through specialized sensory receptors, and kinesthesia, videlicet the sense of motion of the joint [6–8].

 Above-knee amputations result in a loss of a significant number of mechanoreceptors of the knee and generally of the lower limb [9], a fact that consequently leads to proprioceptive deficits. The reduced proprioceptive ability is recorded as a reduced kinesthesia both in the stump and the contralateral nonamputated lower limb [10].

The disruption of the proprioceptive information from the lower limb is followed by central nervous system reorganization, at all levels, from the spinal cord to the cerebral hemispheres, as well as of the afferent and efferent pathways that control and coordinate the movement of the limb [11, 12].

 Significant changes are also observed in balance and gait of patients who suffered from lower limb amputation with asymmetrical weight distribution and shift forward and increased risk of falls [3, 4, 13]. Furthermore, the height of the stump plays decisive role in the gait patterns after amputation. The shorter stump needs greater thrusts during walking and hence greater energy expenditure [4, 14].

 The aim of the present prospective study was to evaluate knee proprioception using the joint position sense (JPS) method, in the limb that underwent above-knee amputation and has an artificial prosthesis for a considerable period of time, and to estimate the adjustments that have been achieved. In this study the predetermined angle of 15° was chosen as the target angle since it is considered to be functional during gait both in stance and the beginning of swing phase [3, 15].

 The evaluation of the data extracted from this study could contribute to better understanding of the complex proprioceptive mechanisms characterizing the study participants. Furthermore it could conduct to the construction of improved artificial prostheses for these patients and to the creation of optimal rehabilitation programmes.

## 2. Materials and Methods

Participants were selected from an initial group of 25 patients with unilateral lower limb amputation. Eventually 22 individuals were recruited, 18 men and 4 women, aged 24–65 years with an average of 42 years (SD: 11.52) who qualified the required criteria (Table 1). 

These patients had undergone amputation above the knee and under the hip and had used artificial prosthesis for at least one year from the amputation [16]. 

Exclusion criteria were bilateral lower or upper limb amputation, problems in auditory and visual system, pain in the stump, alcohol abuse, psychiatric diseases, and the presence of disturbed brain functions or other neurological deficiencies.

Eleven of the patients had undergone an amputation at the lower third of the femoral bone, 7 of them at the medial femur and the remaining 4 at the limit of the medial femur and the lower third of it. 

As regards the cause of the amputation, in 18 patients it was a traffic accident; in 2 of them the presence of malignances and in the remaining one the past history revealed an occupational injury. 

Relative to the type of the prosthesis 10 of them are characterized by hydraulic knee joint system, 10 of them by electronic C-leg, and the other 2 by a simple multiaxial knee joint (Table 1). 

The proprioceptive sensation of the knee was counted with the reproduction of a predetermined angle method (joint position sense) [8, 15, 17], both in the amputated leg and in the healthy leg, which constituted the internal control group. The measurements were performed using a conventional goniometer, more specifically the G300 model, manufactured by Whitehall Manufacturing Hydrotherapy and Health Care Products which has been widely used for clinical evaluation and for research purposes both in healthy populations and in specific groups [18–20]. This goniometer was placed on the center of the artificial knee joint with tape on the prosthesis. 

**Figure 1:**
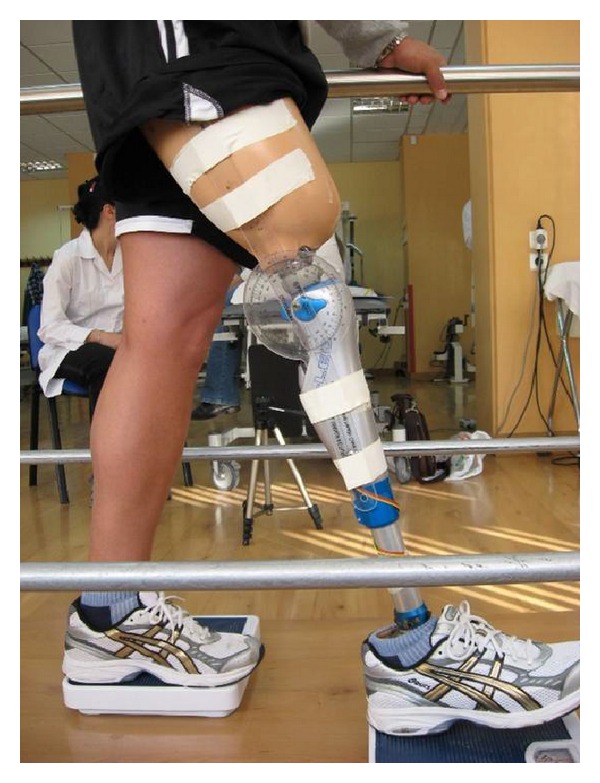
Joint position sense (JPS) evaluation.

According to the width and the length of the patient's step as it was previously determined two analogical scales were placed within the parallel bars in order to specify the exact position of the center of the weight both in the artificial and the normal joint. It was found that the artificial joint carries the center of the gravity in a different place in comparison with the normal knee, and as a result the gravity line stays within the base of support during gait so that the risk of falls will not increase [21–24] (Figure 1). Then, he performed flexion from the position of hyperextension with the artificial knee joint, by increasing the weight on the scale that the artificial limb was leaning. The patient was allowed to use his upper limbs for support. With the researcher's help and the healthy knee hyperextended, the patient brought the under evaluation knee in a flexion angle bigger than 15° for 3 times, so that he could get used to the specific movement. Again with the researcher's help he brought the knee back to the predetermined angle and after a rest that lasted 2 minutes he attempted to reproduce the target angle of the 15° [22, 23, 25]. Three repetitions were performed and the average of the measurements was first calculated and then compared with the corresponding values of the healthy limb in which the aforementioned measurements were previously conducted according to the exact same procedures.

The comparison of the variables between the healthy and the amputated limb was performed using the *t*-test for dependent samples. Data was expressed as mean ± standard deviation (SD) for continuous variables. For the statistical analysis the statistical package SPSS vr 13.00 was used.

All measurements were performed by the same researcher. The reliability of the researcher who conducted the measurements was checked and confirmed. The investigator was requested to measure a specific angle which was ultimately compared to an angle that a second independent researcher was asked to measure. The angles were equivalent and were both specific and unknown to both researchers. 

Both investigators obtained the same data so the correlation between their measurements for the specific angle proved to be strong (correlation coefficient = 1.00) at a statistical significance level of *P* < 0.001.

For the calculation of the measuring angle, apart from the G300 goniometer, a modern isokinetic Con-Trex dynamometer was used, in which a femorotibial functional brace was fitted and the flexion of the knee was performed according to the angle of the isokinetic dynamometer, on the axis that this guide offered.

This study has been approved by the ethics committee of the hospital where it was performed, while all participants granted a written consent that they agreed to be included in the study.

## 3. Results 

Out of 22 patients who participated in the study, 11 of them (50%) had undergone amputation of their dominant leg, while in the remaining 11 the amputation regarded the nondominant leg.

As regards the amputated leg, the values of JSP 15° ranged between 4° and 22.7° with mean 13.91 and SD ±4.74, while for the healthy leg the corresponding values ranged between 10° and 19.7° with mean 14.15° and SD ±2.61° (Table 2).

No statistically significant difference was detected amongst the values of the amputated limbs and the healthy limbs (*t* = 0.258,  *P* > 0.05). 

The measured values in the affected limb differentiated according to the patient's age when he undergone the amputation and the years that he lived with the artificial joint. Patients that used more years the prosthesis presented improved values of proprioceptive sensation.

Moreover statistically significant differences were not recorded in the measured values between the two prevailing those of prosthesis fitted in the study participants (*t* = −0.942,  *P* > 0.05). Specifically the under test patients who used an electronic C-leg joint showed minimum angle 7.7° and maximum angle 22.7° with an average angle M: 15.38° (SD: 4.93°), while those who used hydraulic knee joint presented minimum angle equal to 4° and maximum angle equal to 18° with M: 13.43° (SD: 4.31°) (Table 3).

As regards the reliability of these measurements, it is a considered satisfactory since the value a (coefficient alpha) for the above-mentioned measurements was found greater than 0.80 and precisely equal to 0.95 and 0.97 for the healthy and the operated lower extremity [26]. 

## 4. Discussion

In this research paper, we studied the proprioceptive sensation of the lower limb, with the active reproduction of a predetermined angle method, in a closed kinetic chain environment, using the healthy limb like an internal control group. This angle was selected because it is a representative angle within the functional range of 10° to 60° flexion of the knee, which is required for the normal gait, both for the stance phase and for the beginning of the swing phase [21]. It even seems that the activation of mechanoreceptors is greater in this angle [7, 27].

As it is obvious by the result analysis, a statistically significant difference in the values amongst the limb with the artificial joint and the healthy limb was not reported.

This fact suggests that albeit the study participants showed decreased value of proprioceptive sensation to a certain degree, they did not present a serious decrease of proprioceptive information of the lower limb and more specifically the knee joint, after an above-knee amputation and placement of a prosthesis, for at least one year.

These results are in accordance with the findings of previous researchers, who did not record significant decrease as well, as regards the Joint Position Sense (JPS) after amputation and placement of an artificial joint [15, 17].

Eakin et al. measured the ability of passive reproduction of a predetermined angle in flexion in 5°, 10°, 15°, 20°, and 25°, in an environment of closed kinetic chain. The researchers could not detect significant proprioceptive deficits as regards the target angles and relative to the healthy leg with the previous method. On the contrary, a reduced proprioceptive sensation was recorded in the limb with the prosthesis with the detection of passive motion pathway method [15].

Similar results were recorded by Liao and Skinner in patients with below-knee amputations, who were evaluated for the sense of the joint position in space, and the kinesthetic perception of the knee joint. In that trial not a significant decrease of the proprioceptive information was recorded by measuring the patient's ability to reproduce a predetermined angle with the amputated limb, while even in that study decreased values of kinesthesia characterizing the amputated limb are detected as well [17].

It seems that people with above-knee amputations compensate the loss of the knee joint and the subsequent loss of the mechanoreceptors, via alternative mechanisms that guard the sense of the artificial knee joint.

These mechanisms probably include the hip joint and the cutaneous receptors of the stump which receive impulses from the pressure exerted by the prosthesis [15].

The proprioceptive ability presented increased in this study, when related to the time passed from the amputation and the placement of the prosthesis, a fact that suggests the development of compensatory mechanisms of the loss of the anatomical structures that enhance the proprioceptive sensation of the lower limb as time passes [15].

The role of the adjacent joints in the maintenance of the kinesthetic information seems to be very important. Dhillon et al., in patients with amputation above elbow, at least 4 years ago, reported that the remaining functional connections are viable or able to recover after exercise. Furthermore, sensory neurons appear to be more resistant than motor neurons. Proprioception, even decreased, proved to be capable of recovering even in old amputations [12]. 

Proprioception improvement after a long-term use of the prosthesis appears in the results of this study, as older patients with long-term placement of the prosthesis presented improved values in the measurements.

 In the case of an above-knee amputation there is loss of the knee and of a significant number of mechanoreceptors that support the proprioceptive sensation of the lower limb [9]. Moreover, the proprioception of the quadriceps and the hamstrings is also disturbed [28].

The failure to detect proprioceptive deficits with the method used in this work, unlike other methods that measure kinesthesia, is probably shown due to the different neural mechanisms that are activated during the reproduction of the predetermined angle of 15° flexion used in the herein study [28].

The rehabilitation program followed after an amputation influences the final outcome of the proprioceptive sensation presented in the amputated lower limb. There are numerous physical therapy approaches focusing on different aspects of rehabilitation such as proprioceptive enhancement or restoration of muscle strength. In this study the participants did not follow the same rehabilitation program, a fact that can affect to some extent the results of this work. 

Another weakness of the study is the heterogeneity of the sample as regards the age and the gender of the patients and the fact that the required measurements were performed in a hospital and not in a physical functional environment.

Further randomized trials are required for the exploration of the complex phenomenon of proprioception of the lower extremity after an amputation and placement of an artificial joint, with different methods that measure the different components of proprioception in an environment close, as possible, to normal gait.

## Figures and Tables

**Table 1:** Demographics.

	*N* = 22 m: 18, f: 4 Age: 24–65 (M: 42, SD: 11.52)	
Height of femoral amputation	Cause of amputation	Type of prosthesis
Lower third 11	Traffic accident 18	Hydraulic 10
Medial femur 7	Malignancy 2	C-leg: 10
Medial femur-lower third 4	Occupational injury 1	Multiaxial 2

*N*: number of participants; m: male; f: female; M: mean age; SD: standard deviation.

**Table 2:** Joint position sense. Comparison between health and amputated leg.

	Healthy leg (*N* = 22)	Amputated leg (*N* = 22)	*t*-Test
1	17.3	18	
2	14.7	17.3	
3	10	16.3	
4	15	22.7	
5	18	19.7	
6	10.3	10.3	
7	16	19.7	
8	11.7	11.7	
9	14	12.3	
10	13	7.7	
11	16	20	0.2584
12	12	6.3	
13	16	13.7	
14	19.7	14.7	
15	14	15.3	
16	10	13	
17	15.3	13.3	
18	16.7	11.7	
19	12	16.7	
20	13.7	9	
21	12.7	12.7	
22	13.3	4	
	M: 14.15, SD: ±2.61	M: 13.91, SD: ±4.74	

*N*: number of participants; M: mean age; SD: standard deviation.

**Table 3:** Joint position sense. Comparison between hydraulic and C-leg prosthesis.

Prosthesis	*N*	Mean age	Min.	Max.	M	SD	*t*-Test
Hydraulic	10	45.6	4	13.43	18	4.31	−0.942
C-Leg	10	37	7.7	15.38	22	4.93	

*N*: number of participants; Min.: minimum angle. Max.: maximum angle. M: mean angle; SD: standard deviation.
